# Crystal structure and Hirshfeld surface analysis of (2*E*,2′*E*)-3,3′-(1,4-phenyl­ene)bis­[1-(2,4-di­fluoro­phen­yl)prop-2-en-1-one]

**DOI:** 10.1107/S205698901701564X

**Published:** 2017-11-03

**Authors:** Huey Chong Kwong, Aijia Sim, C. S. Chidan Kumar, Li Yee Then, Yip-Foo Win, Ching Kheng Quah, S. Naveen, Ismail Warad

**Affiliations:** a School of Chemical Sciences, Universiti Sains Malaysia, Penang 11800 USM, Malaysia; b X-ray Crystallography Unit, School of Physics, Universiti Sains Malaysia, 11800 USM, Penang, Malaysia; c Department of Engineering Chemistry, Vidya Vikas Institute of Engineering & Technology, Visvesvaraya Technological University, Alanahally, Mysuru 570028, Karnataka, India; d Department of Chemical Science, Faculty of Science, Universiti Tunku Abdul Rahman, Perak Campus, Jalan Universiti, Bandar Barat, Perak, Malaysia; e Department of Physics, School of Engineering & Technology, Jain University, Bangalore 562 112, India; f Department of Chemistry, Science College, An-Najah National University, PO Box 7, Nablus, West Bank, Palestinian Territories

**Keywords:** crystal structure, hydrogen bond, Hirshfeld surfaces

## Abstract

The asymmetric unit of the title compound, C_24_H_14_F_4_O_2_, comprises of one and a half mol­ecules; the half-mol­ecule is completed by crystallographic inversion symmetry. In the crystal, mol­ecules are linked into a three-dimensional network by C—H⋯F and C—H⋯O hydrogen bonds. Some of the C—H⋯F links are unusually short (< 2.20 Å). Hirshfeld surface analyses (*d_norm_* surfaces and two-dimensional fingerprint plots) for the title compound are presented and discussed.

## Abstract 2

The asymmetric unit of the title compound consists of one and a half bis­chalcone mol­ecules. In the crystal, mol­ecules are linked into a three-dimensional network by C—H⋯F and C—H⋯O hydrogen bonds, some of the C—H⋯F links being unusually short (< 2.20 Å). Hirshfeld surface analyses are presented and discussed.

## Chemical context   

Chalcones, considered to be the precursors of flavonoids and isoflavonoids, are abundant in edible plants. They consist of two aromatic rings joined by a three-carbon-atom unsat­urated carbonyl system (–CH=CH—CO–). Bischalcones with the general formula Ar—CH=CH—CO—CH=CH—Ar (Baeyer & Villiger, 1902 ▸) are an important class of compounds that are widely used in many fields such as organic solid-state photochemistry, and display anti-oxidative and anti-inflammatory activities, cytotoxicity, non-linear optical activity (Uchida *et al.*, 1998 ▸) and fluorescence and luminescent properties (Tay *et al.*, 2016 ▸). Several crystal structures of this type of compound have been reported (Fun *et al.*, 2010 ▸; Park *et al.*, 2013 ▸; Ruanwas *et al.*, 2014 ▸; Sim *et al.*, 2017 ▸). As part of our studies in this area, we report herein the syntheses and structure of the title compound, C_24_H_14_F_4_O_2_, (I), and a Hirshfeld analysis of its inter­molecular inter­actions.
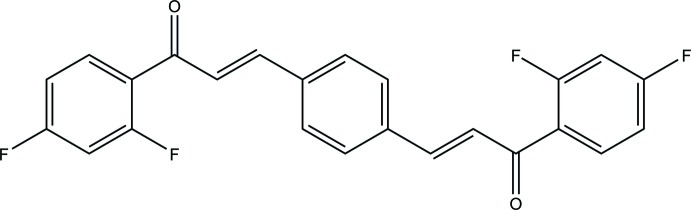


## Structural commentary   

**Figure 1:**
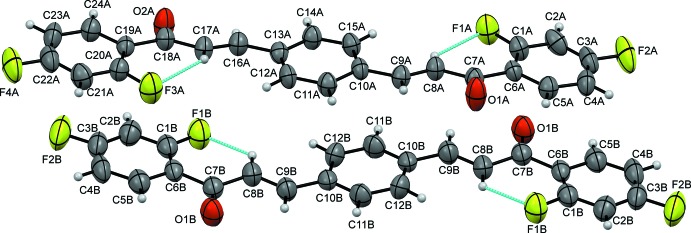
The mol­ecular structure of (I), showing 50% displacement ellipsoids.

The asymmetric unit of (I) with *Z* = ½ consists of one and a half mol­ecules of the bis­chalcone title compound (one complete mol­ecule *A* and a half mol­ecule *B*) (Fig. 1 ▸). The mol­ecule is constructed from two aromatic rings (central benzene and terminal 2,4-di­fluoro­phenyl rings), which are linked by a C=C—C(=O)—C enone bridge, with the carbonyl group in a *cis* conformation with respect to the olefinic double bond. The structural conformation of (I) can be described by three degrees of freedom, which are the torsion angles between the terminal 2,4-difluorophenyl ring and the carbonyl group O1—C7—C6—C1/O2—C18—C19—C20 (τ1); between the carbonyl group and the olefinic double bond O1—C7—C8—C9/O2—C18—C17—C16 (τ2) and between the olefinic double bond and center benzene ring C8—C9—C10—C11/C14—C13—C16—C17 (τ3). In mol­ecule *A*, the carbonyl groups form similar torsion angles with the 2,4-di­fluoro­phenyl ring [O1*A*—C7*A*—C6*A*—C1*A* = −168.4 (4)°; O2*A*—C18*A*—C19*A*—C20*A* = 165.9 (4)°] and the olefinic double bond [O1*A*—C7*A*—C8*A*—C9*A* = −2.1 (5)°; O2*A*—C18*A*—C17*A*—C16*A* = −2.4 (6)°]. Conversely, the torsion angles between the olefinic double bond and the central benzene ring are slightly different [C8*A*—C9*A*—C10*A*—C11*A* = 171.9 (3)°; C14*A*—C13*A*—C16*A*—C17*A* = −166.5 (4)°]. This leads to slight differences in the dihedral angles between the terminal 2,4-di­fluoro­phenyl and the central benzene rings [7.91 (2)° for C1*A*–C6*A* and 6.28 (2)° for C19*A*–C24*A*]. In mol­ecule *B*, both torsion angles τ1 and τ3 are comparable to those in mol­ecule *A* [C1*B*—C6*B*—C7*B*—O1*B* = 171.1 (4)°; C8*B*—C9*B*—C10*B*—C11*B* = 174.2 (4)°]. However, mol­ecule *B* is slightly closer to planar than mol­ecule *A*, as its central and terminal rings subtend a dihedral angle of 5.49 (2)°. This might arise from the lower torsion angle between the olefinic double bond and the central benzene ring [O1*B*—C7*B*—C8*B*—C9*B* = 0.9 (6)°]. Selected torsion and dihedral angles are listed in Table 1 ▸. The C8=C9 double-bond lengths in both mol­ecules are in agreement with expected values reported in the literature (Sathiya Moorthi *et al.*, 2005 ▸).

Each of the intra­molecular C8*A*—H8*A*⋯F1*A*, C17*A*—H17*A*⋯F3*A* and C8*B*—H8*B*⋯F1*B* hydrogen bonds generates an *S*(6) ring motif (Table 1 ▸, Fig. 1 ▸).

## Supra­molecular features   

**Figure 2:**
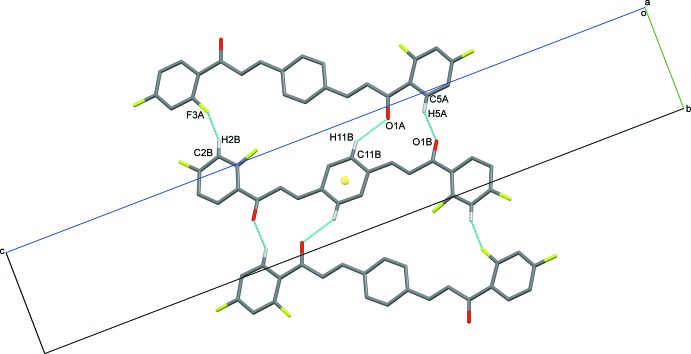
The partial packing of (I), showing a centrosymmetric trimer.

**Figure 3:**
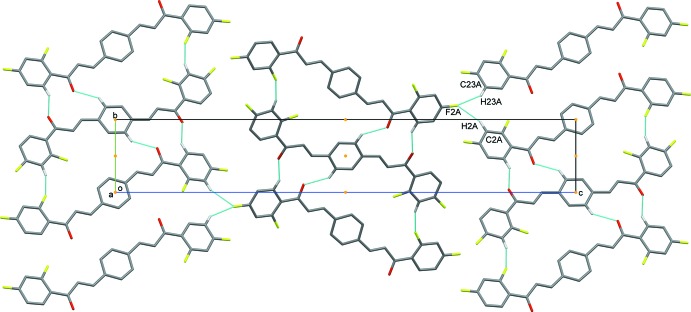
The packing of (I) shown in projection down the *a* axis.

In the crystal of (I), the C11*B*—H11*B*⋯O1*A* hydrogen bonds (Table 1 ▸) generate 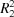
(12) and 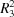
(23) graph-set motifs with the C5*A*—H5*A*⋯O1*B* and C2*B*—H2*B*⋯F3*A* hydrogen bonds (Table 2 ▸). As the central benzene ring of mol­ecule *B* is located about an inversion center, pairs of these hydrogen bonds link the mol­ecules into a centrosymmetric trimer (Fig. 2 ▸, Table 2 ▸). Atom F2*A* acts as double acceptor and links the trimers into a three-dimensional network *via* C2*A*—H2*A*⋯F2*A* and C23*A*—H23*A*⋯F2*A* hydrogen bonds, as shown in Fig. 3 ▸.

## Hirshfeld surface analysis   

The Hirshfeld surface analyses (McKinnon *et al.*, 2004 ▸) of (I) were generated by *CrystalExplorer 3.1* (Wolff *et al.*, 2012 ▸), and can be summarized by fingerprint plots mapped over *d_norm_*. The contact distances to the closest atom inside (*d*_i_) and outside (*d*_e_) of the Hirshfeld surface analyze the inter­molecular inter­action *via* the mapping of *d*_norm_. In a *d*_norm_ surface, any inter­molecular inter­actions will appear as a red spot.

**Figure 4:**
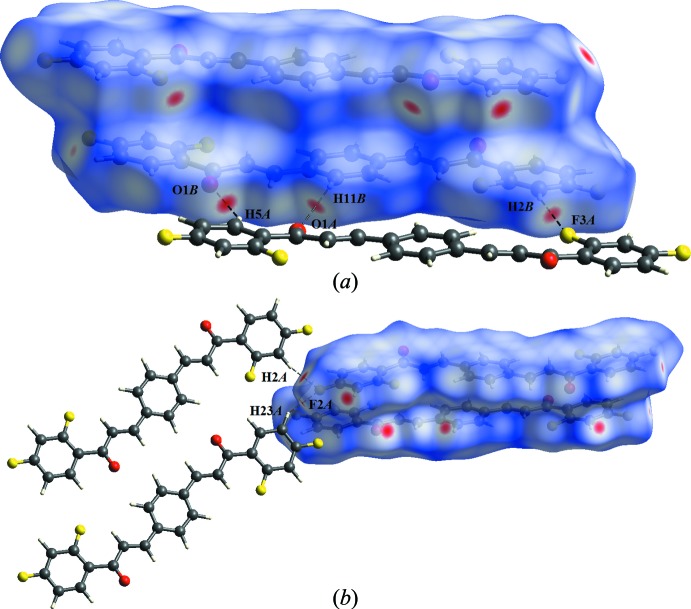
Plots of *d*_norm_ mapped on the Hirshfeld surfaces for (I) showing (*a*) C—H⋯O and C—H⋯F hydrogen bonds and (*b*) C—H⋯F inter­actions.

**Figure 5:**
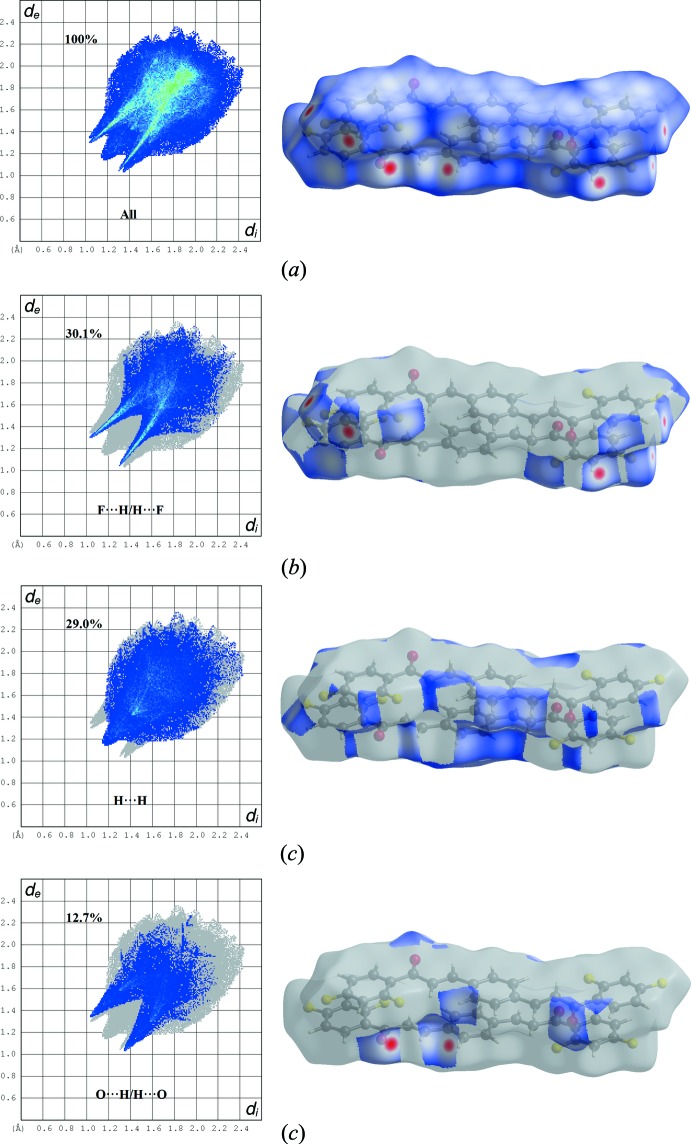
The two-dimensional fingerprint plots for (I) showing contributions from different contacts; views on the right highlight the relevant surface patches associated with the specific contacts.

Dark-red spots that are close to atoms O1*B*, H11*B* and H2*BA* in the *d_norm_* surface mapping are the result of C—H⋯O and C—H⋯F hydrogen bonds (Fig. 4 ▸ *a*). Similarly, the C—H⋯F inter­actions are identified by red spots near the F2*A* atom in mol­ecule *A* (Fig. 4 ▸ *b*). As illustrated in Fig. 5 ▸, the corresponding fingerprint plots (FP) for Hirshfeld surfaces of the title compound are shown with characteristic pseudo-symmetry wings in the *d*_e_ and *d*_i_ diagonal axes represent the overall two-dimensional FP and those delineated into F⋯H/H⋯F, H⋯H and O⋯H/H⋯O contacts, respectively. The most significant inter­molecular inter­actions are the reciprocal F⋯H/H⋯F inter­actions (30.1%), which appear as two sharp symmetric spikes in FP maps with a prominent long spike at *d*_e_ + *d*_i_ ≃ 2.3 Å (Fig. 5 ▸ *b*). The H⋯H inter­actions appear in the central region of the FP with *d_e_* = *d_i_* ≃ 2.4 Å and contribute 29.0% to the Hirshfeld surface (Fig. 5 ▸ *c*) whereas two symmetrical narrow pointed wings corresponding to the O⋯H/H⋯O inter­actions with 12.7% contribution appear at diagonal axes of *d*_e_ + *d*_i_ ≃ 2.4 Å (Fig. 5 ▸ *d*). The percentage contributions for other inter­molecular contacts are less than 10% in the Hirshfeld surface mapping (Fig. 6).

## Database survey   

A search of the Cambridge Structural Database (CSD, Version 5.38, last update Nov 2016; Groom *et al.*, 2016 ▸) using (*E*)-1-(4-fluoro­phen­yl)-3-phenyl­prop-2-en-1-one as the main skeleton revealed the presence of seven structures containing the chalcone moiety with different substituent similar to the title compounds in this study. These structures are 4′-fluoro­chalcone (Ng *et al.*, 2006 ▸), (2*E*)-3-[4-(di­methyl­amino)­phen­yl]-1-(4fluoro­phen­yl)prop-2-en-1-one (Jasinski *et al.*, 2011 ▸), (*E*)-3-(4-chloro­phen­yl)-1-(4-fluoro­phen­yl)prop-2-en-1-one (Fun *et al.*, 2012 ▸), 3-[4-(1*H*-imidazol-1-yl) phen­yl]prop-2-en-1-ones (Hussain *et al.*, 2009 ▸), (*E*)-1-(4-fluoro­phen­yl)-3-(4-methyl­phen­yl)prop-2-en-1-one (Fun *et al.*, 2008 ▸), 1-(4-fluoro­phen­yl)-3-(4-meth­oxy­phen­yl)prop-2-en-1-one (Harrison *et al.*, 2006 ▸) and 3-(biphenyl-4-yl)-1-(4-fluoro­phen­yl)prop-2-en-1-one (Sarojini *et al.*, 2007 ▸). In these seven compounds, the dihedral angles between the central benzene and the fluoro­phenyl rings range from 7.14 to 56.26°.

## Synthesis and crystallization   

A solution of terephthaldi­aldehyde (0.01 mol) in methanol (20 ml) was mixed with 2,4-di­fluoro­aceto­phenone (0.02 mol) in methanol (20 ml) in the presence of NaOH. The reaction mixtures were stirred for about 5–6 h at room temperature. The resultant crude products were filtered, washed successively with distilled water and recrystallized from ethanol solution to get the title compound. Yellow blocks of (I) were obtained by slow evaporation using acetone as solvent.

**(2** ***E*** **,2′** ***E*** **)-3,3′-(1,4-Phenyl­ene)bis­(1-(2,4-di­fluoro­phen­yl)prop-2-en-1-one), C_24_H_14_F_4_O_2_.** Solvent for growing crystals: mixture of chloro­form and aceto­nitrile (1:1 *v*/*v*); yield 85%, m.p. 447–449 K; FT–IR (ATR (solid) cm^−1^): 3101 (Ar, C—H, ν), 1600 (C=O, ν), 1593, 1420 (Ar, C=C, ν), 1229 (C—F, ν); ^1^H NMR (500 MHz, CDCl_3_): δ 7.969–7.922 (*q*, 2H, *J* = 8.7 Hz, ^2^CH), 7.818–7.787 (*d*, 2H, *J* = 15.7 Hz, ^8^CH), 7.697 (*s*, 4H, ^11^CH, ^12^CH), 7.059–7.022 (*t*, 2H, *J* = 8.7 Hz, ^5^CH), 6.969–6.935 (*t*, 2H, *J* = 8.7 Hz, ^4^CH); ^13^C NMR (125 MHz, CDCl_3_): 187.00 (C7), 143.62 (C9), 136.83 (C2), 133.11 (C10), 133.03 (C5), 129.14 (C11, C12), 126.18 (C6), 126.12 (C8) 112.47, 112.27 (C3), 105.01, 104.81 (C1), 104.59 (C4).

## Refinement   

Crystal data, data collection and structure refinement details are summarized in Table 3 ▸. C-bound H atoms were positioned geometrically [C—H = 0.93 Å] and were refined using a riding model with *U*_iso_(H) = 1.2*U*_eq_(C) for H atoms.

## Supplementary Material

Crystal structure: contains datablock(s) I. DOI: 10.1107/S205698901701564X/hb7696sup1.cif

Structure factors: contains datablock(s) I. DOI: 10.1107/S205698901701564X/hb7696Isup2.hkl

Supporting information file. DOI: 10.1107/S205698901701564X/hb7696Isup3.cml

CCDC reference: 1449628

Additional supporting information:  crystallographic information; 3D view; checkCIF report

## Figures and Tables

**Table 1:** Selected torsion and dihedral angles (°) for the title compound The dihedral angle is between the mean planes of the terminal 2,4-di­fluoro­phenyl rings and the central benzene ring.

	Mol­ecule *A*	Mol­ecule *B*
O1—C7—C6—C1/ O2—C18—C19—C20, τ1	−168.4 (4), 165.9 (4)	171.1 (4)
τ2, O1—C7—C8—C9/ O2—C18—C17—C16, τ2	−2.1 (5), −2.4 (6)	0.9 (6)
C8—C9—C10—C11/ C14—C13—C16—C17, τ3	171.9 (3), −166.5 (4)	174.2 (4)
Dihedral angle	7.91, 6.28	5.49

**Table 2:** Hydrogen-bond geometry (Å, °)

*D*—H⋯*A*	*D*—H	H⋯*A*	*D*⋯*A*	*D*—H⋯*A*
C5*A*—H5*A*⋯O1*B* ^i^	0.93	2.49	3.243 (5)	138
C11*B*—H11*B*⋯O1*A* ^ii^	0.93	2.54	3.322 (5)	142
C2*A*—H2*A*⋯F2*A* ^iii^	0.93	2.48	3.362 (5)	158
C2*B*—H2*B*⋯F3*A* ^iv^	0.93	2.50	3.324 (5)	147
C8*A*—H8*A*⋯F1*A*	0.93	2.19	2.822 (4)	124
C8*B*—H8*B*⋯F1*B*	0.93	2.16	2.806 (5)	125
C17*A*—H17*A*⋯F3*A*	0.93	2.19	2.802 (4)	122
C23*A*—H23*A*⋯F2*A* ^v^	0.93	2.56	3.3910	149

Symmetry codes: (i) 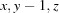; (ii) 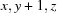; (iii) 
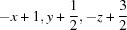
; (iv) 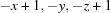; (v) 
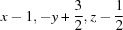
.

**Table 3:** Experimental details

Crystal data	
Chemical formula	C_24_H_14_F_4_O_2_
*M*_r_	410.35
Crystal system, space group	Monoclinic, *P*2_1_/*c*
Temperature (K)	297
*a*, *b*, *c* (Å)	12.190 (6), 5.972 (3), 38.17 (2)
β (°)	98.013 (10)
*V* (Å^3^)	2752 (3)
*Z*	6
Radiation type	Mo *K*α
μ (mm^−1^)	0.12
Crystal size (mm)	0.55 × 0.22 × 0.09

Data collection	
Diffractometer	Bruker APEXII DUO CCD area-detector
Absorption correction	Multi-scan (*SADABS*; Bruker, 2012 ▸)
*T*_min_, *T*_max_	0.870, 0.989
No. of measured, independent and observed [*I* > 2σ(*I*)] reflections	33683, 5127, 3112
*R*_int_	0.053
(sin θ/λ)_max_ (Å^−1^)	0.606

Refinement	
*R*[*F* ^2^ > 2σ(*F* ^2^)], *wR*(*F* ^2^), *S*	0.076, 0.233, 1.09
No. of reflections	5127
No. of parameters	406
H-atom treatment	H-atom parameters constrained
Δρ_max_, Δρ_min_ (e Å^−3^)	0.66, −0.23

Computer programs: *APEX2* and *SAINT* (Bruker, 2012 ▸), *SHELXS97* (Sheldrick, 2008 ▸), *Mercury* (Macrae *et al.*, 2006 ▸), *SHELXL2013* (Sheldrick, 2015 ▸) and *PLATON* (Spek, 2009 ▸).

## supplementary crystallographic information

### Crystal data

**Table:**

C_24_H_14_F_4_O_2_	*F*(000) = 1260
*M**_r_* = 410.35	*D*_x_ = 1.486 Mg m^−^^3^
Monoclinic, *P*2_1_/*c*	Mo *K*α radiation, λ = 0.71073 Å
*a* = 12.190 (6) Å	Cell parameters from 3764 reflections
*b* = 5.972 (3) Å	θ = 2.5–22.7°
*c* = 38.17 (2) Å	µ = 0.12 mm^−^^1^
β = 98.013 (10)°	*T* = 297 K
*V* = 2752 (3) Å^3^	Block, yellow
*Z* = 6	0.55 × 0.22 × 0.09 mm

### Data collection

**Table:**

Bruker APEXII DUO CCD area-detector diffractometer	5127 independent reflections
Radiation source: fine-focus sealed tube	3112 reflections with *I* > 2σ(*I*)
Graphite monochromator	*R*_int_ = 0.053
φ and ω scans	θ_max_ = 25.5°, θ_min_ = 1.7°
Absorption correction: multi-scan (SADABS; Bruker, 2012)	*h* = −14→14
*T*_min_ = 0.870, *T*_max_ = 0.989	*k* = −7→7
33683 measured reflections	*l* = −46→46

### Refinement

**Table:**

Refinement on *F*^2^	0 restraints
Least-squares matrix: full	Hydrogen site location: inferred from neighbouring sites
*R*[*F*^2^ > 2σ(*F*^2^)] = 0.076	H-atom parameters constrained
*wR*(*F*^2^) = 0.233	*w* = 1/[σ^2^(*F*_o_^2^) + (0.1029*P*)^2^ + 1.8579*P*] where *P* = (*F*_o_^2^ + 2*F*_c_^2^)/3
*S* = 1.09	(Δ/σ)_max_ < 0.001
5127 reflections	Δρ_max_ = 0.66 e Å^−^^3^
406 parameters	Δρ_min_ = −0.22 e Å^−^^3^

### Special details

**Table:**

Geometry. All esds (except the esd in the dihedral angle between two l.s. planes) are estimated using the full covariance matrix. The cell esds are taken into account individually in the estimation of esds in distances, angles and torsion angles; correlations between esds in cell parameters are only used when they are defined by crystal symmetry. An approximate (isotropic) treatment of cell esds is used for estimating esds involving l.s. planes.

### Fractional atomic coordinates and isotropic or equivalent isotropic displacement parameters (Å^2^)

**Table:**

	*x*	*y*	*z*	*U*_iso_*/*U*_eq_	
F1A	0.3498 (2)	0.4797 (4)	0.64668 (5)	0.0798 (7)	
F2A	0.5838 (2)	0.1955 (5)	0.74203 (5)	0.1025 (9)	
F3A	−0.0361 (2)	0.5505 (4)	0.34851 (6)	0.0883 (8)	
F4A	−0.2622 (2)	0.8474 (5)	0.25239 (6)	0.1042 (9)	
O1A	0.3887 (3)	−0.1043 (5)	0.59117 (7)	0.0856 (9)	
O2A	−0.0708 (3)	1.1292 (5)	0.40487 (7)	0.0794 (9)	
C1A	0.4176 (3)	0.3132 (6)	0.65981 (8)	0.0540 (9)	
C2A	0.4676 (3)	0.3391 (7)	0.69405 (9)	0.0636 (10)	
H2A	0.4564	0.4672	0.7069	0.076*	
C3A	0.5337 (3)	0.1714 (7)	0.70838 (9)	0.0644 (11)	
C4A	0.5521 (3)	−0.0183 (7)	0.69058 (9)	0.0636 (10)	
H4A	0.5978	−0.1313	0.7011	0.076*	
C5A	0.5008 (3)	−0.0365 (6)	0.65649 (9)	0.0545 (9)	
H5A	0.5125	−0.1654	0.6439	0.065*	
C6A	0.4322 (3)	0.1276 (5)	0.63988 (8)	0.0451 (8)	
C7A	0.3807 (3)	0.0836 (6)	0.60274 (8)	0.0500 (8)	
C8A	0.3236 (3)	0.2583 (6)	0.58072 (8)	0.0502 (8)	
H8A	0.3161	0.4000	0.5902	0.060*	
C9A	0.2820 (3)	0.2184 (6)	0.54740 (8)	0.0506 (8)	
H9A	0.2930	0.0745	0.5392	0.061*	
C10A	0.2221 (3)	0.3691 (5)	0.52217 (8)	0.0459 (8)	
C11A	0.1966 (3)	0.3069 (6)	0.48697 (8)	0.0521 (8)	
H11A	0.2205	0.1685	0.4798	0.063*	
C12A	0.1373 (3)	0.4430 (6)	0.46248 (8)	0.0524 (9)	
H12A	0.1208	0.3946	0.4392	0.063*	
C13A	0.1015 (3)	0.6513 (5)	0.47177 (8)	0.0459 (8)	
C14A	0.1282 (3)	0.7143 (6)	0.50699 (8)	0.0540 (9)	
H14A	0.1058	0.8544	0.5140	0.065*	
C15A	0.1857 (3)	0.5784 (6)	0.53163 (8)	0.0526 (9)	
H15A	0.2008	0.6258	0.5550	0.063*	
C16A	0.0406 (3)	0.8033 (6)	0.44689 (8)	0.0524 (8)	
H16A	0.0359	0.9500	0.4547	0.063*	
C17A	−0.0093 (3)	0.7626 (6)	0.41464 (8)	0.0519 (8)	
H17A	−0.0085	0.6184	0.4055	0.062*	
C18A	−0.0656 (3)	0.9413 (6)	0.39316 (9)	0.0516 (8)	
C19A	−0.1175 (3)	0.9016 (6)	0.35579 (8)	0.0457 (8)	
C20A	−0.1023 (3)	0.7186 (6)	0.33524 (9)	0.0541 (9)	
C21A	−0.1490 (3)	0.6970 (7)	0.30074 (10)	0.0680 (10)	
H21A	−0.1365	0.5710	0.2876	0.082*	
C22A	−0.2141 (3)	0.8657 (8)	0.28658 (9)	0.0682 (11)	
C23A	−0.2331 (3)	1.0523 (7)	0.30478 (10)	0.0684 (11)	
H23A	−0.2779	1.1664	0.2942	0.082*	
C24A	−0.1846 (3)	1.0691 (6)	0.33929 (9)	0.0572 (9)	
H24A	−0.1972	1.1969	0.3521	0.069*	
F1B	0.7643 (2)	−0.0572 (4)	0.60526 (6)	0.0866 (8)	
F2B	0.9643 (2)	−0.2046 (6)	0.71503 (7)	0.1169 (11)	
O1B	0.6556 (2)	0.5348 (5)	0.64536 (7)	0.0775 (8)	
C1B	0.7976 (3)	0.0164 (7)	0.63916 (10)	0.0621 (10)	
C2B	0.8648 (3)	−0.1269 (7)	0.65982 (11)	0.0702 (11)	
H2B	0.8864	−0.2630	0.6511	0.084*	
C3B	0.8987 (3)	−0.0634 (8)	0.69345 (11)	0.0718 (11)	
C4B	0.8691 (3)	0.1350 (8)	0.70704 (10)	0.0749 (12)	
H4B	0.8942	0.1749	0.7303	0.090*	
C5B	0.8008 (3)	0.2739 (7)	0.68516 (9)	0.0667 (10)	
H5B	0.7793	0.4096	0.6940	0.080*	
C6B	0.7627 (3)	0.2187 (6)	0.65007 (8)	0.0538 (9)	
C7B	0.6864 (3)	0.3795 (7)	0.62957 (9)	0.0579 (9)	
C8B	0.6511 (3)	0.3494 (7)	0.59149 (9)	0.0618 (10)	
H8B	0.6755	0.2258	0.5799	0.074*	
C9B	0.5846 (3)	0.4972 (7)	0.57353 (9)	0.0611 (10)	
H9B	0.5626	0.6164	0.5866	0.073*	
C10B	0.5418 (3)	0.4979 (6)	0.53638 (8)	0.0527 (8)	
C11B	0.4812 (3)	0.6741 (6)	0.52103 (9)	0.0623 (10)	
H11B	0.4674	0.7952	0.5351	0.075*	
C12B	0.5603 (3)	0.3208 (6)	0.51443 (10)	0.0627 (10)	
H12B	0.6010	0.1976	0.5237	0.075*	

### Atomic displacement parameters (Å^2^)

**Table:**

	*U*^11^	*U*^22^	*U*^33^	*U*^12^	*U*^13^	*U*^23^
F1A	0.1200 (19)	0.0576 (13)	0.0584 (14)	0.0213 (13)	0.0001 (13)	−0.0063 (10)
F2A	0.132 (2)	0.124 (2)	0.0401 (12)	−0.0345 (18)	−0.0246 (13)	0.0016 (13)
F3A	0.127 (2)	0.0673 (15)	0.0660 (14)	0.0339 (14)	−0.0031 (14)	−0.0057 (11)
F4A	0.122 (2)	0.134 (2)	0.0464 (13)	−0.0110 (18)	−0.0245 (13)	0.0039 (14)
O1A	0.131 (3)	0.0617 (18)	0.0548 (16)	0.0283 (17)	−0.0202 (16)	−0.0127 (14)
O2A	0.113 (2)	0.0579 (17)	0.0584 (16)	0.0191 (16)	−0.0192 (15)	−0.0076 (13)
C1A	0.070 (2)	0.049 (2)	0.0418 (18)	−0.0017 (18)	0.0031 (16)	0.0005 (16)
C2A	0.090 (3)	0.060 (2)	0.0410 (19)	−0.016 (2)	0.0097 (19)	−0.0106 (17)
C3A	0.077 (3)	0.082 (3)	0.0310 (17)	−0.023 (2)	−0.0038 (17)	0.0022 (19)
C4A	0.064 (2)	0.075 (3)	0.047 (2)	−0.004 (2)	−0.0106 (17)	0.0127 (19)
C5A	0.059 (2)	0.056 (2)	0.0466 (19)	0.0003 (17)	0.0006 (16)	−0.0012 (16)
C6A	0.0512 (18)	0.0501 (19)	0.0328 (16)	−0.0030 (15)	0.0023 (14)	0.0046 (14)
C7A	0.060 (2)	0.050 (2)	0.0383 (17)	0.0063 (17)	0.0005 (15)	−0.0045 (15)
C8A	0.060 (2)	0.0465 (19)	0.0408 (18)	0.0020 (16)	−0.0032 (15)	0.0003 (15)
C9A	0.057 (2)	0.0476 (19)	0.0444 (18)	0.0001 (16)	−0.0039 (15)	−0.0014 (15)
C10A	0.0489 (18)	0.0519 (19)	0.0343 (16)	−0.0068 (15)	−0.0033 (14)	0.0043 (14)
C11A	0.063 (2)	0.0482 (19)	0.0425 (18)	0.0012 (16)	−0.0031 (15)	−0.0067 (15)
C12A	0.061 (2)	0.058 (2)	0.0346 (16)	−0.0038 (17)	−0.0060 (15)	0.0001 (15)
C13A	0.0466 (18)	0.0480 (19)	0.0408 (17)	−0.0054 (15)	−0.0018 (14)	0.0023 (14)
C14A	0.064 (2)	0.051 (2)	0.0440 (19)	0.0040 (17)	−0.0023 (16)	−0.0037 (15)
C15A	0.064 (2)	0.055 (2)	0.0359 (17)	−0.0025 (18)	−0.0004 (15)	−0.0037 (15)
C16A	0.059 (2)	0.0490 (19)	0.0463 (19)	0.0025 (16)	−0.0020 (16)	0.0023 (15)
C17A	0.062 (2)	0.0457 (19)	0.0439 (19)	0.0010 (16)	−0.0080 (16)	0.0016 (15)
C18A	0.056 (2)	0.052 (2)	0.0444 (18)	−0.0011 (16)	−0.0018 (15)	0.0006 (16)
C19A	0.0451 (18)	0.0504 (19)	0.0396 (17)	−0.0018 (15)	−0.0013 (14)	0.0051 (14)
C20A	0.057 (2)	0.052 (2)	0.051 (2)	0.0049 (17)	0.0001 (16)	0.0050 (17)
C21A	0.085 (3)	0.069 (3)	0.049 (2)	−0.001 (2)	0.0036 (19)	−0.0074 (19)
C22A	0.070 (2)	0.087 (3)	0.043 (2)	−0.014 (2)	−0.0080 (18)	0.008 (2)
C23A	0.064 (2)	0.076 (3)	0.059 (2)	0.004 (2)	−0.0142 (19)	0.018 (2)
C24A	0.060 (2)	0.055 (2)	0.054 (2)	0.0057 (17)	−0.0013 (17)	0.0044 (17)
F1B	0.1097 (19)	0.0836 (17)	0.0610 (14)	0.0151 (14)	−0.0071 (13)	−0.0051 (12)
F2B	0.100 (2)	0.137 (3)	0.103 (2)	0.0185 (18)	−0.0234 (16)	0.0478 (19)
O1B	0.097 (2)	0.0786 (19)	0.0539 (16)	0.0117 (17)	−0.0005 (14)	−0.0086 (14)
C1B	0.059 (2)	0.078 (3)	0.048 (2)	−0.009 (2)	0.0020 (17)	0.0060 (19)
C2B	0.066 (2)	0.073 (3)	0.070 (3)	0.002 (2)	0.000 (2)	0.012 (2)
C3B	0.061 (2)	0.087 (3)	0.063 (3)	−0.002 (2)	−0.006 (2)	0.024 (2)
C4B	0.075 (3)	0.101 (3)	0.044 (2)	−0.017 (3)	−0.0101 (19)	0.011 (2)
C5B	0.070 (2)	0.076 (3)	0.053 (2)	−0.007 (2)	0.0034 (19)	0.005 (2)
C6B	0.0505 (19)	0.066 (2)	0.0434 (19)	−0.0088 (18)	0.0013 (15)	0.0121 (17)
C7B	0.059 (2)	0.065 (2)	0.049 (2)	−0.0010 (19)	0.0047 (17)	0.0048 (18)
C8B	0.063 (2)	0.075 (3)	0.0450 (19)	0.003 (2)	0.0002 (17)	0.0048 (18)
C9B	0.069 (2)	0.067 (2)	0.046 (2)	0.008 (2)	0.0036 (17)	0.0025 (17)
C10B	0.0471 (19)	0.068 (2)	0.0422 (18)	−0.0011 (18)	0.0040 (15)	0.0049 (17)
C11B	0.076 (2)	0.062 (2)	0.048 (2)	0.010 (2)	0.0078 (18)	−0.0081 (18)
C12B	0.062 (2)	0.063 (2)	0.060 (2)	0.0130 (19)	0.0004 (18)	0.0140 (19)

### Geometric parameters (Å, º)

**Table:**

F1A—C1A	1.344 (4)	C17A—H17A	0.9300
F2A—C3A	1.350 (4)	C18A—C19A	1.497 (4)
F3A—C20A	1.342 (4)	C19A—C20A	1.373 (5)
F4A—C22A	1.358 (4)	C19A—C24A	1.387 (4)
O1A—C7A	1.215 (4)	C20A—C21A	1.366 (5)
O2A—C18A	1.213 (4)	C21A—C22A	1.348 (5)
C1A—C6A	1.370 (5)	C21A—H21A	0.9300
C1A—C2A	1.371 (5)	C22A—C23A	1.350 (6)
C2A—C3A	1.353 (5)	C23A—C24A	1.370 (5)
C2A—H2A	0.9300	C23A—H23A	0.9300
C3A—C4A	1.356 (6)	C24A—H24A	0.9300
C4A—C5A	1.367 (5)	F1B—C1B	1.373 (4)
C4A—H4A	0.9300	F2B—C3B	1.359 (5)
C5A—C6A	1.383 (5)	O1B—C7B	1.195 (4)
C5A—H5A	0.9300	C1B—C2B	1.358 (5)
C6A—C7A	1.492 (4)	C1B—C6B	1.366 (5)
C7A—C8A	1.455 (4)	C2B—C3B	1.347 (6)
C8A—C9A	1.323 (4)	C2B—H2B	0.9300
C8A—H8A	0.9300	C3B—C4B	1.362 (6)
C9A—C10A	1.441 (4)	C4B—C5B	1.372 (5)
C9A—H9A	0.9300	C4B—H4B	0.9300
C10A—C11A	1.387 (4)	C5B—C6B	1.395 (5)
C10A—C15A	1.391 (5)	C5B—H5B	0.9300
C11A—C12A	1.367 (4)	C6B—C7B	1.481 (5)
C11A—H11A	0.9300	C7B—C8B	1.469 (5)
C12A—C13A	1.381 (5)	C8B—C9B	1.323 (5)
C12A—H12A	0.9300	C8B—H8B	0.9300
C13A—C14A	1.390 (4)	C9B—C10B	1.441 (5)
C13A—C16A	1.442 (4)	C9B—H9B	0.9300
C14A—C15A	1.361 (5)	C10B—C11B	1.370 (5)
C14A—H14A	0.9300	C10B—C12B	1.387 (5)
C15A—H15A	0.9300	C11B—C12B^i^	1.378 (5)
C16A—C17A	1.317 (4)	C11B—H11B	0.9300
C16A—H16A	0.9300	C12B—C11B^i^	1.378 (5)
C17A—C18A	1.458 (5)	C12B—H12B	0.9300
			
F1A—C1A—C6A	120.8 (3)	C20A—C19A—C24A	115.6 (3)
F1A—C1A—C2A	116.1 (3)	C20A—C19A—C18A	126.7 (3)
C6A—C1A—C2A	123.1 (3)	C24A—C19A—C18A	117.6 (3)
C3A—C2A—C1A	117.6 (3)	F3A—C20A—C21A	116.2 (3)
C3A—C2A—H2A	121.2	F3A—C20A—C19A	120.1 (3)
C1A—C2A—H2A	121.2	C21A—C20A—C19A	123.7 (3)
F2A—C3A—C2A	118.1 (4)	C22A—C21A—C20A	117.3 (4)
F2A—C3A—C4A	118.8 (4)	C22A—C21A—H21A	121.3
C2A—C3A—C4A	123.1 (3)	C20A—C21A—H21A	121.3
C3A—C4A—C5A	117.2 (4)	C21A—C22A—C23A	123.0 (3)
C3A—C4A—H4A	121.4	C21A—C22A—F4A	118.4 (4)
C5A—C4A—H4A	121.4	C23A—C22A—F4A	118.6 (4)
C4A—C5A—C6A	123.2 (3)	C22A—C23A—C24A	118.2 (4)
C4A—C5A—H5A	118.4	C22A—C23A—H23A	120.9
C6A—C5A—H5A	118.4	C24A—C23A—H23A	120.9
C1A—C6A—C5A	115.8 (3)	C23A—C24A—C19A	122.1 (4)
C1A—C6A—C7A	126.9 (3)	C23A—C24A—H24A	118.9
C5A—C6A—C7A	117.3 (3)	C19A—C24A—H24A	118.9
O1A—C7A—C8A	120.6 (3)	C2B—C1B—C6B	124.7 (4)
O1A—C7A—C6A	117.7 (3)	C2B—C1B—F1B	114.9 (4)
C8A—C7A—C6A	121.8 (3)	C6B—C1B—F1B	120.4 (3)
C9A—C8A—C7A	120.9 (3)	C3B—C2B—C1B	117.1 (4)
C9A—C8A—H8A	119.5	C3B—C2B—H2B	121.4
C7A—C8A—H8A	119.5	C1B—C2B—H2B	121.4
C8A—C9A—C10A	128.3 (3)	C2B—C3B—F2B	118.6 (5)
C8A—C9A—H9A	115.8	C2B—C3B—C4B	123.0 (4)
C10A—C9A—H9A	115.8	F2B—C3B—C4B	118.3 (4)
C11A—C10A—C15A	117.1 (3)	C3B—C4B—C5B	117.7 (4)
C11A—C10A—C9A	120.3 (3)	C3B—C4B—H4B	121.2
C15A—C10A—C9A	122.6 (3)	C5B—C4B—H4B	121.2
C12A—C11A—C10A	121.9 (3)	C4B—C5B—C6B	122.3 (4)
C12A—C11A—H11A	119.0	C4B—C5B—H5B	118.9
C10A—C11A—H11A	119.0	C6B—C5B—H5B	118.9
C11A—C12A—C13A	121.1 (3)	C1B—C6B—C5B	115.2 (3)
C11A—C12A—H12A	119.5	C1B—C6B—C7B	127.7 (3)
C13A—C12A—H12A	119.5	C5B—C6B—C7B	117.0 (4)
C12A—C13A—C14A	116.9 (3)	O1B—C7B—C8B	121.7 (4)
C12A—C13A—C16A	123.3 (3)	O1B—C7B—C6B	117.1 (3)
C14A—C13A—C16A	119.7 (3)	C8B—C7B—C6B	121.3 (4)
C15A—C14A—C13A	122.4 (3)	C9B—C8B—C7B	120.3 (4)
C15A—C14A—H14A	118.8	C9B—C8B—H8B	119.8
C13A—C14A—H14A	118.8	C7B—C8B—H8B	119.8
C14A—C15A—C10A	120.6 (3)	C8B—C9B—C10B	128.5 (4)
C14A—C15A—H15A	119.7	C8B—C9B—H9B	115.7
C10A—C15A—H15A	119.7	C10B—C9B—H9B	115.7
C17A—C16A—C13A	128.9 (3)	C11B—C10B—C12B	116.8 (3)
C17A—C16A—H16A	115.6	C11B—C10B—C9B	121.5 (3)
C13A—C16A—H16A	115.6	C12B—C10B—C9B	121.7 (3)
C16A—C17A—C18A	120.7 (3)	C10B—C11B—C12B^i^	122.6 (3)
C16A—C17A—H17A	119.7	C10B—C11B—H11B	118.7
C18A—C17A—H17A	119.7	C12B^i^—C11B—H11B	118.7
O2A—C18A—C17A	121.0 (3)	C11B^i^—C12B—C10B	120.6 (3)
O2A—C18A—C19A	117.4 (3)	C11B^i^—C12B—H12B	119.7
C17A—C18A—C19A	121.6 (3)	C10B—C12B—H12B	119.7
			
F1A—C1A—C2A—C3A	178.1 (3)	C17A—C18A—C19A—C24A	169.6 (3)
C6A—C1A—C2A—C3A	−0.2 (6)	C24A—C19A—C20A—F3A	178.1 (3)
C1A—C2A—C3A—F2A	179.7 (3)	C18A—C19A—C20A—F3A	0.9 (5)
C1A—C2A—C3A—C4A	−0.2 (6)	C24A—C19A—C20A—C21A	−0.1 (5)
F2A—C3A—C4A—C5A	−179.6 (3)	C18A—C19A—C20A—C21A	−177.3 (3)
C2A—C3A—C4A—C5A	0.3 (6)	F3A—C20A—C21A—C22A	−178.7 (3)
C3A—C4A—C5A—C6A	0.0 (6)	C19A—C20A—C21A—C22A	−0.5 (6)
F1A—C1A—C6A—C5A	−177.8 (3)	C20A—C21A—C22A—C23A	0.8 (6)
C2A—C1A—C6A—C5A	0.4 (5)	C20A—C21A—C22A—F4A	−179.5 (3)
F1A—C1A—C6A—C7A	1.1 (5)	C21A—C22A—C23A—C24A	−0.6 (6)
C2A—C1A—C6A—C7A	179.3 (3)	F4A—C22A—C23A—C24A	179.8 (3)
C4A—C5A—C6A—C1A	−0.3 (5)	C22A—C23A—C24A—C19A	0.0 (6)
C4A—C5A—C6A—C7A	−179.3 (3)	C20A—C19A—C24A—C23A	0.4 (5)
C1A—C6A—C7A—O1A	−168.4 (4)	C18A—C19A—C24A—C23A	177.8 (3)
C5A—C6A—C7A—O1A	10.5 (5)	C6B—C1B—C2B—C3B	0.1 (6)
C1A—C6A—C7A—C8A	12.1 (5)	F1B—C1B—C2B—C3B	−179.2 (3)
C5A—C6A—C7A—C8A	−169.0 (3)	C1B—C2B—C3B—F2B	178.8 (3)
O1A—C7A—C8A—C9A	−2.1 (5)	C1B—C2B—C3B—C4B	−0.3 (6)
C6A—C7A—C8A—C9A	177.4 (3)	C2B—C3B—C4B—C5B	0.4 (6)
C7A—C8A—C9A—C10A	179.0 (3)	F2B—C3B—C4B—C5B	−178.6 (3)
C8A—C9A—C10A—C11A	171.9 (3)	C3B—C4B—C5B—C6B	−0.5 (6)
C8A—C9A—C10A—C15A	−9.3 (6)	C2B—C1B—C6B—C5B	−0.1 (5)
C15A—C10A—C11A—C12A	−0.6 (5)	F1B—C1B—C6B—C5B	179.2 (3)
C9A—C10A—C11A—C12A	178.2 (3)	C2B—C1B—C6B—C7B	−177.5 (4)
C10A—C11A—C12A—C13A	1.0 (5)	F1B—C1B—C6B—C7B	1.8 (6)
C11A—C12A—C13A—C14A	−0.3 (5)	C4B—C5B—C6B—C1B	0.3 (5)
C11A—C12A—C13A—C16A	178.8 (3)	C4B—C5B—C6B—C7B	178.0 (3)
C12A—C13A—C14A—C15A	−0.7 (5)	C1B—C6B—C7B—O1B	171.1 (4)
C16A—C13A—C14A—C15A	−179.8 (3)	C5B—C6B—C7B—O1B	−6.3 (5)
C13A—C14A—C15A—C10A	1.0 (5)	C1B—C6B—C7B—C8B	−9.3 (6)
C11A—C10A—C15A—C14A	−0.4 (5)	C5B—C6B—C7B—C8B	173.3 (3)
C9A—C10A—C15A—C14A	−179.2 (3)	O1B—C7B—C8B—C9B	0.9 (6)
C12A—C13A—C16A—C17A	14.4 (6)	C6B—C7B—C8B—C9B	−178.7 (3)
C14A—C13A—C16A—C17A	−166.5 (4)	C7B—C8B—C9B—C10B	179.5 (4)
C13A—C16A—C17A—C18A	−178.9 (3)	C8B—C9B—C10B—C11B	−174.2 (4)
C16A—C17A—C18A—O2A	−2.4 (6)	C8B—C9B—C10B—C12B	5.6 (6)
C16A—C17A—C18A—C19A	176.7 (3)	C12B—C10B—C11B—C12B^i^	−0.1 (6)
O2A—C18A—C19A—C20A	165.9 (4)	C9B—C10B—C11B—C12B^i^	179.7 (4)
C17A—C18A—C19A—C20A	−13.2 (5)	C11B—C10B—C12B—C11B^i^	0.1 (6)
O2A—C18A—C19A—C24A	−11.3 (5)	C9B—C10B—C12B—C11B^i^	−179.7 (3)

Symmetry code: (i) −*x*+1, −*y*+1, −*z*+1.

### Hydrogen-bond geometry (Å, º)

**Table:**

*D*—H···*A*	*D*—H	H···*A*	*D*···*A*	*D*—H···*A*
C5*A*—H5*A*···O1*B*^ii^	0.93	2.49	3.243 (5)	138
C11*B*—H11*B*···O1*A*^iii^	0.93	2.54	3.322 (5)	142
C2*A*—H2*A*···F2*A*^iv^	0.93	2.48	3.362 (5)	158
C2*B*—H2*B*···F3*A*^v^	0.93	2.50	3.324 (5)	147
C8*A*—H8*A*···F1*A*	0.93	2.19	2.822 (4)	124
C8*B*—H8*B*···F1*B*	0.93	2.16	2.806 (5)	125
C17*A*—H17*A*···F3*A*	0.93	2.19	2.802 (4)	122
C23*A*—H23*A*···F2*A*^vi^	0.93	2.56	3.3910	149

Symmetry codes: (ii) *x*, *y*−1, *z*; (iii) *x*, *y*+1, *z*; (iv) −*x*+1, *y*+1/2, −*z*+3/2; (v) −*x*+1, −*y*, −*z*+1; (vi) *x*−1, −*y*+3/2, *z*−1/2.
